# Effects of Genotype by Environment Interaction on Genetic Gain and Genetic Parameter Estimates in Red Tilapia (*Oreochromis* spp.)

**DOI:** 10.3389/fgene.2017.00082

**Published:** 2017-06-13

**Authors:** Nguyen H. Nguyen, Azhar Hamzah, Ngo P. Thoa

**Affiliations:** ^1^School of Science and Engineering, University of the Sunshine CoastMaroochydore, QLD, Australia; ^2^National Prawn Fry Production and Research CenterKota Kuala Muda, Malaysia; ^3^Research Institute for Aquaculture No.1Tu Son, Vietnam

**Keywords:** red tilapia, harvest weight, genotype by environment interaction, genetic correlation, selection response

## Abstract

The extent to which genetic gain achieved from selection programs under strictly controlled environments in the nucleus that can be expressed in commercial production systems is not well-documented in aquaculture species. The main aim of this paper was to assess the effects of genotype by environment interaction on genetic response and genetic parameters for four body traits (harvest weight, standard length, body depth, body width) and survival in Red tilapia (*Oreochromis* spp.). The growth and survival data were recorded on 19,916 individual fish from a pedigreed population undergoing three generations of selection for increased harvest weight in earthen ponds from 2010 to 2012 at the Aquaculture Extension Center, Department of Fisheries, Jitra in Kedah, Malaysia. The pedigree comprised a total of 224 sires and 262 dams, tracing back to the base population in 2009. A multivariate animal model was used to measure genetic response and estimate variance and covariance components. When the homologous body traits in freshwater pond and cage were treated as genetically distinct traits, the genetic correlations between the two environments were high (0.85–0.90) for harvest weight and square root of harvest weight but the estimates were of lower magnitudes for length, width and depth (0.63–0.79). The heritabilities estimated for the five traits studied differed between pond (0.02 to 0.22) and cage (0.07 to 0.68). The common full-sib effects were large, ranging from 0.23 to 0.59 in pond and 0.11 to 0.31 in cage across all traits. The direct and correlated responses for four body traits were generally greater in pond than in cage environments (0.011–1.561 vs. −0.033–0.567 genetic standard deviation units, respectively). Selection for increased harvest body weight resulted in positive genetic changes in survival rate in both pond and cage culture. In conclusion, the reduced selection response and the magnitude of the genetic parameter estimates in the production environment (i.e., cage) relative to those achieved in the nucleus (pond) were a result of the genotype by environment interaction and this effect should be taken into consideration in the future breeding program for Red tilapia.

## Introduction

Genotype by environment (G × E) interaction refers to the difference in the response of genotypes to different environments (Falconer and Mackay, 1996). There are two main forms of G × E interaction, due to scaling or re-ranking effects. The scaling effect is related to variance difference of traits between environments. A measure of the re-ranking G × E effect is the estimate of genetic correlations between performances in different environments (Falconer, 1952).

For Red tilapia (*Oreochromis* spp.), freshwater pond and cage are two prevailing culture environments in major producing countries, predominantly in Asia (Hamzah et al., 2008; Pongthana et al., 2010). In addition, a range of other production systems has been practiced including canals, mining pools, lakes, and reservoirs (Hamzah et al., 2008). In our breeding program for this species, selection was practiced for body weight in freshwater ponds and performance testing of siblings from each family was also conducted in freshwater cages over a grow period of about 3–4 months (Hamzah, unpublished). Recently, Thodesen et al. (2013) reported, in a different population of red tilapia, that the genetic correlation between body weight in freshwater earthen ponds and floating cages was high (0.92 ± 0.06), while that between freshwater earthen ponds and brackish water tanks was low (0.33 ± 0.14). The G × E interaction effect was reported for a range of fish species, including Nile tilapia (Bentsen et al., 2012; Trọng et al., 2013), rainbow trout (Kause et al., 2003), Atlantic cod (Kolstad et al., 2006), Asian and European sea bass (Dupont-Nivet et al., 2010; Domingos et al., 2013; Le Boucher et al., 2013). A synthesis of the literature review across farmed aquaculture species indicates that when the environments are similar (e.g., pond vs. cage), the G × E effect is not of commercial importance for body traits (e.g., Nguyen, 2016; Sae-Lim et al., 2016). However, when the environments in questions are remarkably dissimilar (e.g., freshwater vs. brackish water as reported in the study of Luan et al., 2008), the G × E interaction is significant especially for traits that are largely influenced by environmental factors, such as survival, sexual maturity, or fitness related traits.

To date, the extent to which genetic gain was realized in production systems (e.g., cage) when selection was conducted in a different environment (i.e., pond) is still not estimated in red tilapia. Quantification of such differences in the genetic gain and genetic parameter estimates between the testing and selection environments in the nucleus is needed because the aquaculture sector particularly for red tilapia is characterized by a diverse array of production systems from backyard farming to semi-medium and intensive large scales (Eknath et al., 2007; Trọng et al., 2013). In extreme cases if the G × E is significant, this should call the conduct of separate breeding programs for each production environment (Nguyen and Ponzoni, 2006; Sae-Lim et al., 2016). Understanding magnitude of the G × E effects on genetic gain and genetic parameter estimates (heritability and correlations) would assist not only the design but also the optimization of commercial genetic improvement programs for this species (Mulder et al., 2006; Sae-Lim et al., 2016).

The principal aim of our study was, hence, to evaluate selection response for four body traits in the two tested environments, namely pond and cage. In addition, we examined the magnitude of genotype by environment interaction for traits studied and reported the genetic parameters for body traits and survival in pond and cage culture.

## Materials and methods

### The genetic selection line

The genetic selection line was established in 2008 from 103 full-sib families (one male × one female), each represented by 20–50 fish produced from a complete diallel cross involving three founder stocks (two imported stocks originated from Taiwan and Thailand, and another one from top ranking animals in a strain evaluation of 20 hatchery stocks in Malaysia; Hamzah et al., 2008). The founder stocks were reared under the same pond culture environments until they reached an average body weight of about 250–400 g before mating was initiated. In 2009, the progeny of the diallel cross experiment was produced in Malaysia, thus creating what we call the base population (G0). The selection line was formed with the 2009 progeny produced from 79 sires and 79 dams and it was selected for high breeding value for harvest body weight. A combined between and within family selection was practiced. The average proportion of selected animals was 3.0% in females and 1.6% in males. Selection was practiced on breeding values for body weight but not by truncation (due to inability to reproduce of some selected breeders we had to resort to selecting lower ranking ones; also, the number of selected individuals contributed by each family was restricted to a maximum of two males and four females in order to avoid later inbreeding and mating of black spot fish was avoided. On average, the progeny was produced in each generation from 46 to 79 males and 53 to 79 dams. This design and experimental size were adhered throughout the course of the selection program (2010–2012) during which the data used for this study were collected and reported here.

### Family production

In each generation, production of full- and half-sib families was conducted in hapas installed in ponds, following the nested mating design (one male × two females) prepared from annual routine genetic evaluation and mate allocation analyses. However, in practice, the two females were not always successfully mated with a male, due to breeding failure or mortality loss. After 7 days of mating, fertilized eggs were collected from the mouth of the female and immediately transferred to hatching jars for artificial incubation. Fry were often hatched after about 5–7 days. As soon as after sac-yolk absorption, the hatched fry of each family were transferred from the incubators to the nursery hapas (1 m^3^ with 2 mm mesh size), stocked at a density of 200 fry per m^3^. At least three nursery hapa replicates for each family were maintained in the same pond to reduce environmental differences between families. When the fingerlings reached an average weight of 5–10 g, about 100–150 individuals per family were randomly sampled and physically identified, using PIT (passive integrated transponder) tags. At tagging, the identification number, body weight (BW), standard length (L), body depth (D), and body width (W) were also recorded. After conditioning for about 3 days in fiberglass tanks without feeding, the tagged fingerlings were pooled together and representatives of each family were sent to communal grow-out testing in freshwater earthen pond and cage. The same producers were repeated in all generations during the course of the selection program for Red tilapia.

### Testing environments and data recording

The tagged fingerlings from each full-sib family were randomly allocated to two testing environments in cages or earthen ponds. Six cages (3.5 m width × 3.5 m length × 3 m depth) were positioned adjacent to each other in an irrigation canal (water depth of about 1.5 m) at Kodiang, Kedah, 22 km away from Jitra. An equal number of sibs per family was randomly assigned to each cage with an initial stocking density of 5 fish per square meter of surface water. The feeding rate was from 3 to 5% of their body weight on a commercial dry pellet feed with 32% protein content twice a day. Contemporaneously with freshwater cage culture, siblings from all families were also tested in earthen ponds of 0.01 ha located at the Aquaculture Extension Center, Department of Fisheries, Jitra, Kedah. The density in each pond was 2–3 fish per square meter of surface water. The same feeding, culturing and management practices were applied as used for the cages. In both environments, water quality parameters (temperature, pH, dissolved oxygen and total ammonia) were monitored once a week.

At the end of the grow-out period, fish were harvested after pond drainage to measure body traits and survival. Individual fish was weighted using a digital scale (nearest to 0.1 g). Standard length was also measured with a ruler. Body width and depth were measured at the mid-side of the fish, where they were largest, by an electronic digital caliper (0.01 mm). Survival was recorded as a binary response (coded as 1 for fish that were present at harvest and 0 for those were absent). Body weight data collected at harvest was then processed to determine estimated breeding values (EBVs) for all individuals in the pedigree. The best animals (highest EBV for body weight) were selected to become parents of the next generations. The same procedures regarding the family production, genetic evaluation and selection process were repeated in subsequent generations during the course of the study from 2009 to 2012. Table 1 shows the pedigree structure (number of sires, dams, and progeny) recorded over four generations from 2009 to 2012 (including the base population) in the two testing environments (i.e., freshwater pond and cage).

**Table 1:** Number of sire, dam and progeny in pond and cage environments.

**Environment**	**Spawning year**	**Line**	**Sire**	**Dam**	**Progeny**
Pond	2009	Base population	79	79	7,283
	2010	Selection	46	55	2,375
	2011	Selection	43	50	2,080
	2012	Selection	52	69	3,265
	Sub-total		220	253	15,003
Cage	2009	Base population	–	–	–
	2010	Selection	35	38	1,366
	2011	Selection	39	44	1,344
	2012	Selection	45	60	2,203
	Sub-total	Selection	119	142	4,913
Total			224	262	19,916

*Total number of common sires and dams in both environments*.

### Statistical analysis

Statistical analyses were performed on 19,916 data records (15,003 fish in ponds combined with 4,913 fish in cages) collected over three generations of selection (2010–2012), tracing back to the base population in 2009. Basic statistics given in Table 2 show that the fish were harvested at a similar weight and size in both pond and cage environments. All traits were evaluated for normality before undertaking further analyses and raw data were transformed when appropriate. Exploratory analyses using a general linear model (GLM) and PROC UNIVARIATE (SAS Institute Inc., 2007) were undertaken. All body traits followed approximate normal distribution, but square root transformation of body weight was also used to improve normality of the residuals. A series of statistical analyses was conducted separately for each environment as follows:

**Table 2:** Number of observations (n), mean, standard deviation (SD) and coefficient of variation (CV) for traits studied in in pond and cage environments.

**Environment**	**Traits**	**Unit**	***n***	**Mean**	**SD**	**CV,%**
Pond	Weight	g	4,206	245.7	96.7	39.4
	Length	cm	4,206	18.3	3.5	19.4
	Width	cm	4,206	7.7	1.4	18.7
	Depth	cm	4,206	3.4	0.7	20.6
	Survival	%	4,206	55.0	47.8	
Cage	Weight	g	2,122	251.4	138.5	55.1
	Length	cm	2,122	17.6	2.9	16.6
	Width	cm	2,122	7.9	1.7	21.7
	Depth	cm	2,121	3.2	0.7	21.3
	Survival	%	2,122	43.8	49.6	

### Univariate analysis using linear mixed model

After all the significant fixed effects were identified; the full model with the family effects was applied to analyse all traits studied. In matrix notation the mixed model can be written as:

(1) y=Xb+Za+Wc+e

Where *y* is the vector of observations for body traits and individual survival, *b* is the vector of the fixed effects including generation (or spawning seasons, 1–3), sex (female or male) and the two-way interactions between generation and sex. A linear covariate (age from birth to harvest) was fitted within sex by generations subclass as well-stocking weight (*P* < 0.05). Vector *a* is the random animal additive genetic effects ~ (0, A $\sigma_{a}^{2}$) where A is the additive genetic (numerator) relationship matrix among the animals, c is the vector of family effects (or maternal effects in addition to additive genetics) ~ (0, I $\sigma_{c}^{2}$) and *e* is the vector of residual effects ~ (0, I $\sigma_{e}^{2}$). The dam component ($\sigma_{D}^{2}$) is most likely a combination of maternal and common environmental effects (thus, $\sigma_{D}^{2}=\sigma_{M+CE}^{2}$, referred to as $\sigma_{C}^{2}$) caused by the separate rearing of full-sib families until individuals reached a suitable size for physical tagging. The log likelihood ratio test showed that the *c*^2^ effects were significant for all the traits (*P* < 0.05). X, Z, and W are incidence matrices relating observations to fixed effects, additive genetic effect of the individual animal and common effects to full-sib included in the model, respectively.

The mixed model equation for the best linear unbiased estimator (BLUE) of estimable functions of b and the best linear unbiased prediction of a and c are:

(2) $$\left[\begin{array}{l} \hat{b}\\ \hat{a}\\ \hat{c} \end{array}\right]={\left[\begin{array}{ccc} \text{X}\prime \text{X} & \text{X}\prime \text{Z} & \text{X}\prime W\\ \text{Z}\prime \text{X} & \text{Z}\prime \text{Z}+{\text{A}}^{-1}\alpha_{1} & \text{Z}\prime W\\ W\prime \text{X} & W\prime \text{Z} & W\prime W+\text{I}\alpha_{2} \end{array}\right]}^{-1}\left[\begin{array}{r} \text{X}\prime y\\ \text{Z}\prime y\\ W\prime y \end{array}\right]$$

where $\alpha_{1}=\sigma_{e}^{2}/\sigma_{a}^{2}and\alpha_{2}=\sigma_{e}^{2}/\sigma_{c}^{2}$.

Under the linear mixed model [1], heritabilities for body traits and survival were calculated as $h^{2}=\frac{{\hat{\sigma }}_{a}^{2}}{{\hat{\sigma }}_{a}^{2}+{\hat{\sigma }}_{c}^{2}+{\hat{\sigma }}_{e}^{2}}$ and the common full-sib effect as $c^{2}=\frac{{\hat{\sigma }}_{c}^{2}}{{\hat{\sigma }}_{a}^{2}+{\hat{\sigma }}_{c}^{2}+{\hat{\sigma }}_{e}^{2}}$ where $\sigma_{a}^{2}$ is the additive genetic variance, the maternal variance ($\sigma_{c}^{2}$) and the residual variance ($\sigma_{e}^{2}$).

### Generalized linear mixed model

In addition to the linear mixed model as described above (Equation 1), survival was analyzed by generalized linear mixed model (GLMM), assuming that the data followed a binomial and underlying normal distributions with a logit link function (McCulloch et al., 2008). Under the logit model, the link function ($\hat{p}$ = e^x^/(1 +e^x^)) was used where *p* is the probability of fish survival recorded at harvest. The fixed effects were the same as defined in Equation (1), but the random effects were sire (*s*_*k*_) and common full-sib group (*c*_*l*_). With this logit threshold model, heritability was calculated using the variance of the logit link function, which implies a correction of the residual variance by factor π^2^/3.

(3) $$h^{2}=\frac{4\sigma_{s}^{2}}{\sigma_{s}^{2}+\sigma_{c}^{2}+\sigma_{e}^{2}\frac{\pi^{2}}{3}}$$

where $\sigma_{s}^{2}$ is sire variance, $\sigma_{c}^{2}$ is common full-sib variance and $\sigma_{e}^{2}=1$.

For binomial observations, estimates of *h*^2^ on the observed (0/1) scale can be transformed to the underlying liability scale (logit) using the formula of Robertson and Lerner (1949) as follows:

(4) $$h_{L}^{2}=\frac{h_{O}^{2}p(1-p)}{z^{2}}.$$

where $h_{O}^{2}$ is the heritability on the observed (0/1) scale, $h_{L}^{2}$ is the estimated heritability on the liability (logit or probit) scale, *p* is a proportion of survival in the data, and *z* is the height of the ordinate of normal distribution corresponding to a truncation point applied to *p* proportion of those traits. A similar transformation was also made for *c*^2^.

### Multivariate analysis

#### Genetic correlation for the same trait between pond and cage environments

Multivariate linear mixed model was applied to estimate the genetic correlation between homologous traits in pond and cage environments. In these analyses, body measurements in each environment were treated as different traits in order to examine whether there were differences associated with culture environment. The model was as described above, also note that the fixed effect of environment was not included. Genetic correlations between expression of body traits in pond and cage were estimated via numerator genetic relationships matrix **(A)** in the full pedigree. Since the traits were measured on animals in different environments, there is no environmental covariance between them. The phenotypic correlations also do not exist because any individual fish will only express the trait in one environment.

#### Genetic and phenotypic correlations among traits in each environment

Multivariate linear mixed model analyses were also used to estimate the genetic and phenotypic correlations separately for each environment. The correlations were calculated from a series of bivariate analysis as the covariance divided by the product of the standard deviations of traits: $r=\frac{\sigma_{12}}{\sqrt{\sigma_{1}^{2}}\sqrt{\sigma_{2}^{2}}}$ where $\sigma_{12}$ was the estimated additive genetic or phenotypic covariance between the two traits, and $\sigma_{1}^{2}$ and $\sigma_{2}^{2}$ are the additive genetic or phenotypic variances of traits 1, 2, respectively. The multivariate analyses included final (harvest) body weight to minimize any possible bias in genetic parameter estimates (Kennedy, 1990).

All the statistical analyses were conducted in ASReml, including the full pedigree traced back to the base population.

### Direct selection response and correlated responses

The direct response to selection for body weight in pond environment was measured as changes in estimated breeding value (EBVs) using the full animal model (Equation 1). This model was also used to estimate the correlated responses in standard length, body depth, body width, and survival in cage and pond environments. The same fixed effects as shown in Equation (1) were used in all analyses. The EBV estimates for body weight and other traits were expressed in an actual unit (g for weight, cm for length, width and depth, and % for survival) and in genetic standard deviation units (estimated breeding values in actual unit/the square root of genetic variance).

## Results

### Genetic parameters in each culture environment

#### Heritabilities

Table 3 presents the variance components, heritability and common environmental effects for harvest weight, total length, body width, body depth, and survival in pond and cage environments. In either pond or cage environment, the estimated heritabilities for body traits were low to moderate, ranging from 0.05 to 0.39. Square root transformation of body weight slightly increased magnitude of the heritability in cage environment, whereas the estimate remained unchanged in pond. This is likely due to the improved distribution of the residuals in the latter (cage) relative to the former (pond) environments. Heritability for survival estimated from linear and nonlinear (generalized) mixed model using logit link function was low in pond (0.02–0.18), whereas the estimates in cage were moderate to high (0.19–0.68) (Table 3). When the observed heritability of linear survival (model 1) was transformed to the underlying liability scale, the *h*^2^ estimates also differed between pond (0.013) and cage (0.119). The common full-sib effects (or maternal and common environmental effects, *c*^2^) for body traits and survival were relatively large and the estimates were higher in pond than in cage (0.23 to 0.51 vs. 0.13–0.31, respectively).

**Table 3:** Variance components, heritability (*h*^2^) and common full-sib effects (*c*^2^) for body traits in pond and cage environments.

**Environment**	**Traits**	**V_*A*_**	**V_*C*_**	**V_*E*_**	***h*^2^**	***c*^2^**
Pond	Weight^0.5^	2.16	6.14	2.02	0.21 ± 0.10	0.59 ± 0.05
	Weight	1727.3	3920.6	2122.4	0.22 ± 0.10	0.51 ± 0.05
	Length	1.54	2.87	7.86	0.13 ± 0.08	0.23 ± 0.05
	Width	0.026	0.20	0.34	0.05 ± 0.08	0.36 ± 0.05
	Depth	0.37	0.83	0.69	0.19 ± 0.10	0.44 ± 0.06
	Survival^A^	0.0051	0.075	0.175	0.02 ± 0.06	0.29 ± 0.04
	Survival ^B^	0.075	0.61	1	0.18 ± 0.13	0.33 ± 0.04
Cage	Weight^0.5^	1.33	1.43	4.36	0.19 ± 0.12	0.20 ± 0.06
	Weight	563.9	1141.7	5312.4	0.08 ± 0.08	0.16 ± 0.05
	Length	0.29	1.40	2.77	0.07 ± 0.10	0.31 ± 0.06
	Width	0.095	0.037	0.12	0.39 ± 0.13	0.15 ± 0.06
	Depth	0.52	0.19	0.72	0.36 ± 0.13	0.13 ± 0.06
	Survival^A^	0.037	0.030	0.129	0.19 ± 0.08	0.15 ± 0.04
	Survival ^B^	2.40	1.70	1	0.68 ± 0.18	0.11 ± 0.03

A , Observed scale (0/1) and

B *, Liability scale using logit function*.

*V_A_, V_C_, and V_E_, Genetic, common full-sib and residual variances, respectively*.

*Weight^0.5^, Square root transformation*.

### Correlations among traits studied in each environment

Phenotypic and genetic correlations among traits studied in pond and cage environments are presented in Table 4. In general, the genetic correlations among body traits including live weight, total length, body width, and depth in cage were positive and higher than the phenotypic correlations in pond. The genetic correlations among body traits studied were high and close to unity in both pond and cage environments. The harvest weight showed non-significant genetic correlations with survival due to the high standard errors, although the estimates were positive in pond and negative in cage. Similarly, the phenotypic correlations between survival and body traits were in opposite directions between the two environments (Table 4). This was likely due to the greater mortality loss in cage than in pond and the dead fish in cage were those which had “good” size (big, thick, and round body shape). The genetic correlation between harvest weight and survival may have varied with the environments used or population-specific.

**Table 4:** Phenotypic (above) and genetic (below) correlations among traits in pond and cage environments.

**Environment**		**Weight**	**Length**	**Width**	**Depth**	**Survival**
Pond	Weight		0.65 ± 0.02	0.63 ± 0.03	0.84 ± 0.01	−0.01±.04
	Length	0.99 ± 0.03		0.43 ± 0.03	0.59 ± 0.02	0.18 ± 0.05
	Width	0.90 ± 0.11	0.94 ± 0.12		0.79 ± 0.02	0.32 ± 0.39
	Depth	0.96 ± 0.03	0.99 ± 0.03	0.98 ± 0.03		0.17 ± 0.04
	Survival	0.54 ± 0.51	0.21 ± 0.15	−0.23±.32	0.16 ± 0.29	
Cage	Weight		0.80 ± 0.01	0.70 ± 0.02	0.82 ± 0.01	−0.21 ± 0.04
	Length	0.92 ± 0.13		0.73 ± 0.02	0.81 ± 0.01	0.92 ± 0.04
	Width	0.99 ± 0.14	0.95 ± 0.14		0.74 ± 0.02	−0.75 ± 0.09
	Depth	0.97 ± 0.04	0.97 ± 0.05	0.99 ± 0.04		−0.55 ± 0.03
	Survival	−0.73 ± 0.88	0.75 ± 0.56	0.04 ± 1.98	0.15 ± 1.56	

### Between-environment genetic correlations for body traits

The genetic correlations between trait expressions in pond and cage environments were high (0.62 to 0.90; Table 5). The between-environment genetic correlation estimates for harvest weight were greater than those obtained for other body traits (*r*_*g*_ = 0.90 vs. 0.62–0.79). The estimated genetic correlations for length and survival between pond and cage environments were high but they were associated with large standard error (*r*_*g*_ ± SE = 0.63 ± 0.41 and 0.70 ± 0.61, respectively).

**Table 5:** Genetic correlations between trait expressions in pond and cage environments.

**Traits**	**Genetic correlation**	**S.E**
Weight^0.5^	0.85	0.14
Weight	0.90	0.10
Length	0.63	0.41
Width	0.79	0.18
Depth	0.62	0.18
Survival	0.70	0.61

### Selection responses in pond and cage environments

Genetic gain measured as EBVs and expressed in an actual unit (g) and in a genetic standard deviation unit (σ_A_) is given in Table 6 for pond and cage environments. Note that selection was performed in pond and the indirect response was measured in cage. The direct and correlated responses estimated in this population were all in desired directions. Overall, the direct selection and correlated responses were greater in pond than those in cage, except for correlated changes in body width. The genetic trend increased steadily with generations of selection for all traits studied in both environments (pond and cage; Table 6). The magnitude of the selection response obtained for body weight in square root transformation scale was in good agreement with those estimated on original scale of measurement (0.390 vs. 0.310 σ_A_). Selection for increased harvest body weight resulted in positive correlated genetic changes in survival rate with the gain of 0.567 and 1.561 genetic standard deviation units in cage and pond after three generations, respectively.

**Table 6:** Genetic responses measured as estimated breeding values in actual unit (g) and in genetic standard deviation unit (σ_A_) in pond and cage environments.

**Env**.	**Years**	**Genetic gain in harvest weight**				**Correlated responses in body traits and survival**							
		**Weight**^**0.5**^		**Weight**		**Length**		**Depth**		**Width**		**Survival**	
		**Actual**	**σ_A_**	**Actual**	**σ_A_**	**Actual**	**σ_A_**	**Actual**	**σ_A_**	**Actual**	**σ_A_**	**Actual**	**σ_A_**
Pond	2010	0.003	0.002	0.215	0.005	0.017	0.014	0.002	0.004	0.000	−0.003	0.022	0.308
	2011	0.568	0.387	17.784	0.428	0.270	0.218	0.157	0.257	0.017	0.104	0.071	0.996
	2012	1.188	0.809	34.686	0.835	0.479	0.386	0.337	0.554	0.035	0.216	0.111	1.561
Cage	2010	−0.036	−0.031	−0.871	−0.037	−0.011	−0.020	−0.024	−0.033	−0.007	−0.023	0.027	0.138
	2011	0.220	0.191	4.746	0.200	0.049	0.092	0.122	0.200	0.044	0.273	0.068	0.351
	2012	0.450	0.390	7.365	0.310	0.102	0.190	0.302	0.497	0.133	0.825	0.109	0.567

*The estimates for harvest weight in cage were the correlated responses to selection in pond*.

## Discussion

In Red tilapia, a prevailing culture system in commercial production is freshwater cage. In contrast, selection programs in the nucleus are often conducted in pond. The difference between the production and selection environments may have impacts on genetic gain and population parameter estimates. Our findings showed that the correlated responses in freshwater cages and the direct gain obtained in earthen ponds differed between pond (0.011–1.156 σ_A_) and cage (−0.033 to 0.567 σ_A_) environments. The gain in body weight was greater in pond than in freshwater cage across generations of selection from 2010 to 2012. The reduced selection response in cage relative to pond was likely due to the G × E interaction, which consists of two forms: scaling (heterogeneity of the variances) or re-ranking effects (low genetic correlation estimate for the same trait between the two environments). The scaling effect on genetic gain was generally not important (Ponzoni et al., 2008; Sae-Lim et al., 2016). Hence, the differences in the selection response in the present study may have been a result of the re-ranking effect. A measure of the re-ranking G × E effect is the genetic correlation for harvest weight between pond and cage environments; the estimate, albeit high, was not close to one. This is also because selection was practiced in pond not in cage or due to different environmental factors between the two culture systems. The smaller number of fish were tested in the latter (i.e., cage) than the former environment (pond) and the mortality rate as well fish loss due to diseases or environmental factors were greater in cage than in pond. It was likely due to the poor water quality in the irrigation canal or due to the limited space in cages. However, other factors may have been involved with the high mortality in cage, e.g., social competition. Overall, the selection response obtained for harvest weight in the present study was comparable with those reported in a different population of Red tilapia (Thodesen et al., 2013), Nile tilapia (Ninh et al., 2014; de Oliveira et al., 2016), common carp (Dong et al., 2015), marine finfish (Knibb et al., 2016) and/or giant freshwater prawn (Hung et al., 2013).

In a similar trend to body weight, correlated responses in other body traits (standard length, width, and depth) and survival were also greater in pond than in cage culture. The correlated genetic changes achieved for body traits were all in desired directions. The changes in body traits suggest that the Red tilapia line grew faster in the pond compared with the cage, whereas the fish reared in the cage was thicker than those in pond. Trọng et al. (2013) also observed that the Nile tilapia grown in cage develop a thicker body shape at the same length compared to fish grown in ponds. Further, the small positive correlated changes in survival were achieved in both environments, although its genetic associations with body weight were not significant, due to the high standard errors. This also reflects that correlation is not causation (cause and effect), especially for traits that are largely influenced by environmental factors as observed for survival in this study.

The discrepancy in the selection responses between the production and nucleus in this population of Red tilapia is consistent with the genetic correlation estimates between homologous traits recorded in freshwater pond and cage culture environments, which are different from one, especially for length, width and depth. Our estimates of the between-environment genetic correlations were, however, in accordance with those published for a range of different culture environments for harvest weight in Nile tilapia (Eknath et al., 2007; Bentsen et al., 2012; Khaw et al., 2012; Thodesen et al., 2013; Trọng et al., 2013; Thoa et al., 2016). In other farmed aquaculture species, the genetic correlation estimates reported for harvest weight between contrasting environments during grow-out were also very high and close to one, such as in Rainbow trout (Sylvén et al., 1991; Fishback et al., 2002; Kause et al., 2003), Atlantic cod (*Gadus morhua*) (Kolstad et al., 2006), Whitefish (*Coregonus lavaretus*; Quinton et al., 2007), Barramundi (Domingos et al., 2013), European sea bass (Dupont-Nivet et al., 2010; Le Boucher et al., 2013), Pacific white shrimp (*P. vannamei*) (Gitterle et al., 2005) and Oysters (*Crassostrea gigas*) (Swan et al., 2007). On the contrary, a low genetic correlation (*r*_*g*_ = 0.45 ± 0.09) between freshwater and salt water environments has been reported in Nile tilapia by Luan et al. (2008). A similar finding was also reported by Mas-Muñoz et al. (2013) for harvest weight (*r*_*g*_ = 0.56) and specific growth rate (*r*_*g*_ = 0.27) between intensive recirculation aquaculture system (RAS) and semi-natural environment (POND) in common sole (*Solea solea*), Arctic charr (*Salvelinus alpinus*) by Chiasson et al. (2013) and rainbow trout (Sae-Lim et al., 2013). These authors suggested that there was a strong genotype by environment interaction between these diverse environments used in their studies.

In addition to the selection responses and the G × E effects, the estimates of heritability for body traits and survival differed between pond and cage environments. Square root transformation used in this study reduced the magnitude of the difference in the heritability estimates for body weight between the two environments. The slight difference in heritability between grow-out testing environments was also reported in Nile tilapia or other fish species, such as Artic char (Chiasson et al., 2013) and rainbow trout (Sae-Lim et al., 2013). However, a meta-analysis of 26 studies in various aquaculture species indicated that the effect size of the difference in heritability for body weight between contrasting environments was not significant, *P* > 0.05 (Nguyen, unpublished results). Further, when analyses were conducted separately for each environment, the genetic correlation estimates among body traits were almost identical (close to one). The genetic correlations between harvest weight and survival, although not significant, had opposite signs between ponds and cages. Phenotypically, weight, width and depth had negative correlations with survival, likely because dead fish in cages are those which had “good” (thick and round) body shape. Overall our estimates of heritability and genetic correlations for body traits and survival are in line with the published results in Nile tilapia (Thoa et al., 2016) or rainbow trout (Sae-Lim et al., 2015).

On the other hand, the common full-sib effects (*c*^2^) obtained for body traits in the present study were greater than those reported for other fishes (Nguyen et al., 2007) or crustacean species (Hung et al., 2014). This was mainly due to the separate rearing of each family in hapas for about 2 months before the fish were physically tagged to enable the communal grow-out of all families in ponds and cages. The *c*^2^ effects were also larger in ponds than cages, which can be explained because of the management and environmental differences between the two culture systems.

Collectively, our results suggest that even though when the genetic correlations between trait expressions in environments are high, the amount of selection response would provide practical information to determine whether the G × E interaction is of biological significance or not. This is because in the context of aquaculture, the G × E interaction can occur, due to the large variation between the selection and production environments in terms of culture environments (Bentsen et al., 2012), scales of production (e.g., small vs. large commercial operations; Trọng et al., 2013), agro-climatic conditions (Sae-Lim et al., 2013, 2017), as well various farming and husbandry management practices (Eknath et al., 2007; Hamzah et al., 2014; Ninh et al., 2014).

In summary, a combination of the genetic parameter estimates and the realized selection responses achieved in the present study pointed out that there may be a need to conduct separate genetic evaluation of selection response for pond and cage culture in Red tilapia. However, multiple genetic improvement programs for different environments may not be fully justified when the predicted losses in production are smaller than the cost of running a new program (Nguyen, 2016). In farmed aquaculture species, a single breeding program is virtually always implemented for a range of environments especially in developing countries where resources and experience in managing breeding programs are limited. This is also due to a biosecurity issue, i.e., preventing disease transmission from commercial production populations into the nucleus. One strategy we practiced to minimize G × E effects in the present breeding program was that relatives of selection candidates were performance tested in both pond and cage, and a combined genetic evaluation of the data recorded in both environments alleviated the G × E effects, thus lessening the loss in genetic gain. Optimization of the selective breeding programs is also discussed by Sae-Lim et al. (2016).

## Conclusion

The selection responses captured for body traits in the production environment (cage) were lower than those achieved in the selection environment (i.e., earthen ponds). The genetic correlation between pond and cage environments for body traits including harvest weight (the selection criterion), albeit high, was not close to one. There were also differences in the heritability and common full-sib effects between the two environments. However, running separate breeding programs for each environment is hardly justified, due to our limited resources. In the future genetic improvement for this population of Red tilapia, selection index approach should be applied by treating the expression in each environment as a separate trait, and weighing the traits in proportion to the relative importance of pond and cage culture. Accounting for the genotype by environment effects in the selective breeding program of Red tilapia will contribute to the sustained development of the aquaculture sector in major tilapia producing countries world-wide.

## Ethics statement

The study and the protocol were reviewed and approved by the animal ethics committee of the Department of Fisheries, Malaysia (the approval number ANE070312). They were standard commercial procedures.

## Author contributions

Conceived and designed the study: NN, and AH. Managed and oversaw the project: NN and AH. Analyzed the data and wrote the paper: NN, AH, and NT.

### Conflict of interest statement

The authors declare that the research was conducted in the absence of any commercial or financial relationships that could be construed as a potential conflict of interest.
